# Factors Affecting Pre-Hospital and In-Hospital Delays in Treatment of Ischemic Stroke; a Prospective Cohort Study

**DOI:** 10.22037/aaem.v9i1.1267

**Published:** 2021-07-24

**Authors:** Neda Ghadimi, Nasrin Hanifi, Mohammadreza Dinmohammadi

**Affiliations:** 1 School of Nursing and Midwifery, Zanjan University of Medical Sciences, Zanjan, Iran.

**Keywords:** Ischemic stroke, Time-to-Treatment, Prospective studies, Iran

## Abstract

**Introducion:**

The outcomes of acute ischemic stroke (AIS) are highly affected by time-to-treatment. The present study aimed to determine the factors affecting in-hospital and pre-hospital delays in treatmentof AIS.

**Methods:**

This prospective study was carried out on 204 AIS patients referring to the stroke care unit in Zanjan (Iran) in 2019. The required data were collected by interviewing the patients and families and using patients’ records and observations.

**Results:**

The maximum delay was related to onset-to-arrival time (288.19 ± 339.02 minutes). The logistic regression analysis indicated a statistically significant decline in the treatment delay via consultation after the initiation of symptoms (p< 0.001), transferring the patient through emergency medical service to the hospital (p<0.001), and patients’ perception regarding AIS symptoms (P< 0.001).

**Conclusion:**

It is essential to inform people regarding AIS symptoms and referring to AIS treatment units to reduce the treatment time.

## 1. Introduction:

Stroke is one of the most prevalent neurological complications (1). Acute ischemic stroke (AIS) is a medical emergency that requires intensive treatment and care in the early hours, because its fast diagnosis and proper interventions can lead to favorable results. Furthermore, delayed treatment can lead to considerable complications, higher mortality, and enormous costs for the person, families, and the healthcare system (2).

The most effective approaches to treating AIS patients are recanalization and reestablishing blood flow to the brain tissues using invasive and non-invasive therapies (3). In these processes, the blocked vessels are reopened using recombinant tissue plasminogen activator (rTPA) and mechanical devices (angioplasty) (4, 5). In 1996, the Food and Drug Administration (FDA) recommended using rTPA in AIS patients within the first 3 h of symptoms onset (6). American Stroke Association Standard (2018) recommends brain imaging within less than 20 min, the interval of less than 60 min between the hospital arrival, and thrombolytic therapy for over 50% of patients qualified for rTPA (7). 

Research has shown that rTPA is avoided as it wastes the golden time of medication use due to the delayed arrival at the hospital (8). The time-to-treatment delay in AIS patients may be caused by different factors such as pre-hospital and intra-hospital reasons. Delays in recognizing and transfer of patients are among the pre-hospital causes of their mortality. Meanwhile, delays in neurologic visits, delays in decision-making regarding the treatment procedure, and delays in brain imaging are considered among the intra-hospital delay causes (9, 10). Treatment delay is a function of several factors, including the patient’s delay after the onset of early symptoms and delay by treatment staff (11, 12). Lack of access to medical centers (13) and lack of proper management of AIS patients in the hospital are among the factors influencing the time-to-treatment delay (14, 15). In this respect, Code 724 has been effective in reducing the delay in treating AIS patients (16). 

In Iran, the stroke code (Code 724) was announced by Iran’s Ministry of Health to the medical universities in 2016 to treat stroke patients more effectively. Thus, in addition to implementing this plan, it is essential to review the status of pre-hospital and hospital delays in Stroke Care Units (SCU) in Iranian cities. In every community, it is essential to investigate the factors influencing pre-hospital and in-hospital delay, the quality of care delivered, and the individual factors affecting timely treatment. These influencing factors can vary from community to community. The present study aimed to determine the factors influencing in-hospital and pre-hospital delays in treatment of AIS.

## 2. Methods:

***2.1 Study design and setting***

This cross-sectional descriptive study was performed in the SCU of Vali-Asr Hospital, Zanjan (northwest of Iran), from July to the end of October 2019. AIS cases or their relatives were interviewed about potentioal causes of delay in initiation of thrombolytic therapy using a predesigned questionnaire (appendix 1). The Ethics Committee of Zanjan University of Medical Sciences approved this study under the Ethics Code IR.ZUMS.REC.1398.095. The researcher described the study’s aims to the patients or their families, and written consent was obtained. The participants were assured about the confidentiality of all their information and the right to leave the study at any time.

***2.2 Participants***

The samples were collected using convenience sampling. Therefore, the study participants included patients referring to the SCU during the sampling interval, who met the inclusion criteria. The physician confirmed the diagnosis of AIS based on clinical signs and brain CT-scan results. Willingness to participate in the study was considered the inclusion criterion. Patients diagnosed with a hemorrhagic stroke or transient ischemic attack (based on CT-scan results) were excluded from the study.

***2.3 Procedure***

SCU of Vali-Asr Hospital in Zanjan, was established in 2016 and is known as the stroke referral center in Zanjan Province. In Iran, Code 724 refers to stroke patients whose stroke symptoms have initiated less than 4 hours and 30 minutes before. Based on this code, as soon as the patients call the Emergency Medical Services (EMS), they are asked about the Face-Arms-Speech-Time (FAST) symptoms. Then, after the ambulance is sent to the patient’s bedside, the emergency technician examines the FAST symptoms, and need to SCU is reported followed by confirmation. Patients from the neighboring provinces are immediately transferred from all medical centers to the SCU in Zanjan. After transferring the patient to the hospital, a neurologist examines them at the triage unit and sends him/her to the brain computed tomogramphy (CT) scan if the diagnosis of a stroke is made based on the scan, the rTPA medication is administered there.

***2.4 Data gathering***

The variables in this study included demographic characteristics, factors affecting the time of treatment initiation in both in-hospital and pre-hospital phases, and stroke risk factors (hypertension, hyperlipidemia, smoking, and diabetes). 

A questionnaire was used to collect the data and identify the information on demographic features and factors affecting time-to-treatment and the average time between onset of symptoms to treatment (7, 11, 17-20). The questionnaire included three parts. Questions about the demographic features of the patients were included in the first part. The second part contained questions regarding the causes of pre-hospital delays. The third part included questions about the reasons for in-hospital delay (Appendix 1). These data were approved by the treating physician. To assess AIS severity, we considered the National Institutes of Health Stroke Scale (NIHSS) (21). This scale includes 11 items, for which a score of 0 denotes the individual’s normal performance in the studied field, and a score of 4 represents maximum impairment in that field. The maximum and minimum scores on this scale are 42 and 0, respectively. In this regard, the score 0 denotes lack of stroke symptoms, 1 to 4 is mild stroke, 5-15 is moderate stroke, 16-20 is moderate to severe stroke, and 21-42 denotes severe stroke. 

The content validity of the questionnaire was evaluated. The designed questionnaire was offered to 10 experts to make the essential modifications and alterations they believed to be necessary. Its reliability was assessed using inter-rater relaibility. Two researchers completed the questionnaire for the same 10 patients, simultaneously. Then, Cohen’s kappa coefficient was assessed between the data of the researcher-completed questionnaires, and the evaluators’ reliability was confirmed by achieving K = 0.973. The reliability and validity of the NIHSS tool had been confirmed by Kasner et al. (21).

The data were collected through observation and interviews with patients and their families, if necessary. The patients referring to the SCU were chosen based on the inclusion criteria. The researcher completed the questionnaire after treatment and relative stabilization of the patient with the assistance of the patient or his/her caregivers. In this study, to reduce recall bias regarding the timing of the events by the patients and their families, and recording the times and factors influencing pre-hospital delays as accurately as possible we highlighted the critical times like news time, Azan time, and events of the day when asking about the events .

**2.5 Data analysis**

According to a pilot study on 20 AIS patients, we considered a sample size of 181, an effect size of 0.05, a sampling error of 20 min, and a confidence level of 95%. In this study, 204 patients with AIS referring to the SCU were assessed.

Statistical analyses were performed using SPSS V.16 software. The data were distributed based on the normalized central limit theorem. Data were gathered through interviews and observations. For detecting the predictors of delay to treatment, a logistic regression model was performed using the Forward-LR method. To determine the factors affecting time-to-treatment, variables including age (age less than 60 years and age over 60 years), gender, previous history of stroke, calling EMS, consultation after the onset of symptoms, and patient’s perception of early symptoms were entered to the model as independent variables. In contrast, delay in treatment was used as the dependent variable. In this study, the significance level was considered less than 0.05. There was no missing data in the present study, because the researchers collected data through interviews, observations, and patient records.

## 3. Results:

***3.1 Baseline characteristics of participants***

This study was conducted on 230 patients with stroke referring to the SCU from early July to late October 2019. The data of 16 patients with transient ischemic attack and 10 patients with hemorrhagic stroke were excluded from the study. Ultimately, the data of 204 patients with acute ischemic stroke who had referred to the SCU were assessed. The treating physician diagnosed the ischemic stroke in these patients.

In total, 204 patients were included in this study, 55.9% of which were male, 19.6% had a high school diploma, and 72.5% were illiterate. The participants’ mean age was 68.99 ±13.91 (28 - 98) years. Fifty percent of the patients lived in Zanjan. According to patients’ statements, 87.7% had at least one risk factor. Hypertension (59.3%) was the most prevalent risk factor for AIS, and ischemic heart disease was in the second rank (30.4%). Moreover, about 77.9% of the patients were at home when the symptoms had initiated. The severity of the stroke was moderate in 52% of the patients. In this study, 140 (68.6%) patients were referred to the SCU with code 724. They arrived at the hospital within less than 4 hours (h) and 30 minutes (min) after the onset of symptoms. Moreover, rTPA was provided for 129 (63.2%) patients, but it was not used for 75 (36.8%) patients. 

***3.2 Analysis of delay to treatment***

The reason for not receiving rTPA in 64 (31.4%) patients was that more than 4 hours and 30 minutes had passed from the symptoms’ onset to referral to SCU. Table 1 shows the frequency of potentioal prehospital causes of delay in treatment of AIS cases. 70.6% of the patients considered their prime symptoms to be symptoms of other diseases and did not believe they had a stroke. Furthermore, 17.2% had no consultation with anyone after the onset of the symptoms and took no action. After the onset of symptoms, about 47.5% of the patients referred to medical centers rather than SCU. It is noteworthy that they mostly (30.4%) referred to these centers because of availability or proximity. 46.1% of them referred to SCU using personal vehicles. A neurologist performed the first visit for more than half of the patients (62.7%). The mean onset-to-arrival time and the mean onset-to-treatment time were 288.19 ± 339.02 minutes and 314.13 ± 341.04 minutes, respectively. 

Table 2 shows the time interval between onset of symptoms and treatment based on pre-hospital and in-hospital factors. Among the pre-hospital delay factors, the delay in deciding to contact the emergency service or making the effort to refer to medical centers (204.74 ± 321.38 minutes) was longer compared to the time of patient transfer to the hospital (83.52 ±72.38 minutes).

In identifying the predictors of delay in treatment, among the predictor variables included in the model, calling EMS, patient’s perception of early symptoms, and consultation after the onset of symptoms could effectively predict this delay. The odds of decreasing the delay in treatment for transportation by EMS, patient’s perception of early symptoms, and consultation after the onset of symptoms were 0.12 (95%CI: 0.033-.435), 7.46 (95%CI: 2.04-27.3), and 0.008 (95%CI: 0.001-0.05), respectively (table 3).

## 4. Discussion:

Our results indicated that pre-hospital delay was longer compared to the hospital delay. The delays in making the effort to refer to the medical center or the decision to call the emergency service were longer compared to the time of patient transfer to the hospital.

In a study in Hamadan (Iran), Ghiasian et al. reported that the time interval between symptom onset to arrival at the hospital was 282 min, while it was 192 min in the study of Griesser et al (11, 22). This result is consistent with the findings of our study. Nevertheless, in the study of Ayromlou et al. in Tabriz (Iran), this time was 916 min, which is not in line with our results (13). In the mentioned study, which was conducted in the metropolitan area of Tabriz, the delay in patients’ arrival could be caused by traffic problems in this city. In the smaller towns around the provinces equipped with SCUs, accurate diagnosis of the stroke, the existence of neurologists, and administering thrombolytic medication can dramatically decrease the onset-to-treatment time.

Koksal et al., Ruiz et al., Faiz et al., Sobral et al., Springer et al., and Haiqiang et al. showed that access to transfer with EMS shortens the delay in hospital arrival (12, 17, 19, 23-25). In our study, also, less delay was experienced by the patients referring via EMS.

Consistent with our study, the findings of studies conducted in America, Asia, and Europe indicated that absence of awareness of stroke symptoms, patients’ beliefs and misconceptions about the prime symptoms, and failure to consult an individual after the onset of the symptoms resulted in longer delays in hospital arrival and time-to-treatment for stroke patients (11, 12, 17, 19, 22-27). The results indicate that consulting with others after initiation of the symptoms may help prevent a delay in cases the symptoms of the patients are not well-recognized or taken seriously.

The results of our investigation on factors causing hospital delay in AIS patients revealed that there were no delay for AIS patients receiving Code 724. In this study, the time interval between hospital arrival to rTPA implementation (25.18 ±17.01 min) and between hospital arrival to brain CT scan (10.60 ± 6.79 min) was much shorter compared to the time proposed by the American Stroke Association guidelines (7). In the study by Dhaliwal et al. in the US, the mean initial CT scan time was 13.66 min, the CT scan interpretation time was 25.20 min, and the time between the arrival of the patients and rTPA injection was 51.27 min (15). Hasankhani et al. in Tabriz (Iran) found that the mean time between hospital arrival and rTPA injection is 69 min (14). In the study of Mowla et al. in New York, the maximum imaging delay was longer than 25 min (28). According to the findings obtained in Iran and other countries, the time interval between hospital arrival and treatment in patients with Code 724 is much longer compared to our results. This indicates that the management of the stroke code team in Zanjan city have been able to significantly shorten time-to-treatment.

**Table 1:** The frequency of potential pre-hospital causes of delay in initiation of treatment for patients with acute ischemic stroke

Number (%)	**Variables**
**Patient’s perception of early symptoms**	
60 (29.4)	Neurologic
144 (70.6)	Non-neurologic
**Consultation after the onset of symptoms**	
49 (24.0)	Spouse
94 (46.0)	Children
4 (2.0)	Colleague
18 (8.8)	Relatives
4 (2)	Nurse
35 (17.2)	Not consulting anyone
**Center visited after symptom onset**	
107 (52.5)	SCU in Zanjan
55 (27.0)	Other medical centers
28 (13.6)	Clinic
13 (6.4)	Private office
1 (0.5)	Private hospital
**Reasons for not referring toSCU**	
62 (30.4)	Proximity or availability of another center
8 (3.9)	Not being aware of stroke center at SCU
27 (13.2)	Not considering the disease seriously by the patient
	**Referred to hospital by**
94 (46.1)	Personal vehicle
64 (31.4)	Emergency medical Services (EMS)
44 (21.5)	Ambulance from other medical centers
1 (0.5)	Stroke inside the hospital
1 (0.5)	Air emergency
**The first visitor of the patient**	
2 (1.0)	General Practitioner
128 (62.7)	Resident of Neurology
68 (33.3)	Emergency medicine specialist
5 (2.5)	Neurologist
1 (0.5)	Non-neurology Resident

SCU: stroke care unit.

**Table 2:** The time interval between onset of symptoms and treatment based on pre-hospital and in-hospital factors

**Mean ± SD**	**Variables**
	**Pre-hospital time intervals** ** (minutes)**
204.74 ± 321.4	Onset –to- decision time
83.52 ± 72.4	The transfer time
288.19 ± 339	Onset –to- arrival time
	**In-hospital** **time intervals ** **(minutes)**
3.07 ± 2.5	Door –to –examination time for with Code 724
15.08 ± 8.5	Door –to –examination time for without Code 724
17.99 ± 13.1	Door –to –SCU entry time for with Code 724
216.98 ± 173.5	Door –to –SCU entry time for without Code 724
10.6 ± 6.9	Door –to –imaging time for with Code 724
21.87 ± 13.9	Door –to –treatment decision making for with Code 724
23.08 ± 16.5	Door–to –order time for with Code 724
25.01 ± 17	Door –to –needle time for with Code 724
29.07 ± 33.8	Door –to – treatment time for without Code 724
314.13 ± 341	Onset –to –treatment time in stroke patients

SD: standard deviation; SCU: stroke care unit.

**Table 3:** Indipendent predictors of delay in treatment of acute ischemic stroke cases

**Logistic regression analysis**						**Variables**
**EXP(B)**	**P**	**df**	**Wald**	**S.E**	**B**	
0.008	0.001	1	24.468	917	4.536	Consultation after the onset of symptoms
0.12	0.001	1	13.433	0.646	2.369	Transportation by EMS
7.46	0.003	1	8.532	0.536	-1.565	Patient’s perception of early symptoms
8.92	0.001	1	19.886	0.627	-2.796	Constant

B=Beta, S.E= Standard Error, df=degrees of freedom, EXP (B)= Ecpected Beta; *P-value< 0.05.

## 5. Limitation

The low accuracy of recalling the times, particularly in elderly patients, was among the limitations of this study. The researchers tried to record the times and factors influencing pre-hospital delays as accurately as possible by highlighting the critical times like news time, Azan time, and events of the day. Considering the geographical and cultural position of Zanjan, the present results cannot be generalized to other communities.

## 6. Conclusion:

In the present study, a longer pre-hospital delay was found compared to hospital delay in stroke events. Among the pre-hospital delay factors, the delay in visiting a medical center or deciding to call the EMS was longer than the time of patient transfer to the hospital. In other words, a more significant portion of the delays in the pre-hospital phase is caused by the delay in patients’ decision to refer to the hospital. It appears that giving information to at-risk people, particularly those over 60 years, about the stroke risk factors, the importance of rapidly initiating treatment to enhance the disease outcomes, and the early stroke symptoms will help patients comprehend their symptoms properly. Hence, they will be transferred to the hospital faster by calling the emergency system.

## 7. Declarations:

### 7.1 Acknowledgment

This study is based on a research project with the code A-11-148-19. Here, the researchers thank the research participants for their cooperation and Zanjan University of Medical Sciences Vice-Chancellor for financial support.

### 7.2 Authors' contributions

NH designed the study, carried out statistical analyses of the data, was involved in interpreting the data, and wrote the manuscript. NG, who also collected the data, was involved in the interpretation of the data. MR D was involved in the interpretation of the data. All authors read and approved the final manuscript.

### 7.3 Funding and supports

This article results from a Master of Nursing thesis funded by the research department of Zanjan University of Medical Sciences.

### 7.4 Competing interests

The authors declare that they have no competing interests.

### 7.5 Pre print this article

Part of this article on the site Research Sequare is online as pre print.

Available in: https://www.researchsquare.com/article/rs-135115/v1
